# Improving interoperability between microbial information and sequence databases

**DOI:** 10.1186/1471-2105-6-S4-S23

**Published:** 2005-12-01

**Authors:** Paolo Romano, Peter Dawyndt, Francesca Piersigilli, Jean Swings

**Affiliations:** 1 Bioinformatics and Structural Proteomics, National Cancer Research Institute, Largo Rosanna Benzi 10, I-16132, Genova, Italy; 2 Laboratory of Microbiology, Ghent University, K.L. Ledeganckstraat 35, B-9000 Ghent, Belgium; 3 Department of Mathematics and Computer Science, University of Camerino, Camerino (MC), I-62032, Italy; 4 BCCM™/LMG Bacteria Collection, Ghent University, K.L. Ledeganckstraat 35, B-9000 Ghent, Belgium

## Abstract

**Background:**

Biological resources are essential tools for biomedical research. Their availability is promoted through on-line catalogues. Common Access to Biological Resources and Information (CABRI) is a service for distribution of biological resources and related data collected by 28 European culture collections. Linking this information to bioinformatics databanks can make the collections' holdings more visible after a search in molecular biology databanks and vice-versa. Identification of links to sequence databases can be useful, but annotation and indexing problems, together with compilation errors, immediately arise. In this paper, we present our efforts for the identification of cross-references between CABRI catalogues and the EMBL Data Library and related results.

**Results:**

An SRS site with both EMBL and CABRI catalogues has been set up. Ad-hoc changes in indexing scripts allowed to achieve homogeneous index keys and SRS link features have been used to identify links between databases. After manual checking and comparison with an alternative procedure, about 67,500 valid cross-references were identified, added to the EMBL Data Library and are now distributed with it. HTML links can be established from EMBL to CABRI network service. Procedures can be executed whenever needed.

**Conclusion:**

Links between EMBL and CABRI catalogues constitute an improved access to micro-organisms of certified quality and can produce positive effects on biomedical research. Further links between CABRI catalogues and other bioinformatics databases can now easily be defined by using these cross-references. Linking genetic information onto natural resources information may stand model for the integration of other databases containing empirical data on these materials.

## Background

Biological resources, including microbial strains, human and animal cell lines, plasmids, phages, plant cells and plant cell viruses, are essential tools for today's biomedical research. They are collected in specialized centres where they are adequately characterized and stored. Centres also work in agreement with guidelines for high quality management of these resources, including their transfer and distribution under proper conditions. Availability of biological resources is promoted by means of purpose catalogues which are now commonly available on the web and/or on CD-ROMs. Catalogues report detailed descriptions of the resources. This information is not only intended to identify single elements of the collection, but also to fully describe their biological properties and special functions. Often, catalogues include bibliography references that can support scientists to reach further information. The main limitation to accessibility of this information is that catalogues are usually available in separate web sites and scientists are therefore obliged to search many of them in order to find the resource that is the most adequate to their research.

Common Access to Biological Resources and Information (CABRI) [1,2] is a network service for the distribution of resources and related data that are collected and managed by a number of European culture collections. Through the CABRI service, more than 110,000 biological resources from 28 collections (see table 1) can be searched in the same site and through a single query. Searching abilities include queries by scientific name and by strain number and free text search. Search by scientific name includes an option for adding synonyms' support. Through CABRI services, resources can also be pre-ordered on-line by using an electronic shopping cart. In this way, a tight connection between collections and scientists is achieved.

Linking information that is available in culture collections' catalogues to bioinformatics databanks that are available on the Internet can further significantly improve the accessibility of biological resources and can make the collections' holdings effectively more visible after a search in molecular biology databanks and vice-versa. As a consequence, additional certified information can be made available to researchers and a growing number of them will hopefully refer to culture collections and make a wider use of biological material of certified quality. In this context, extensions of catalogues' information are being carried out at many centres, depending on their research interests. Efforts were also done in the frame of the European Biological Resource Centres Network (EBRCN) project [3], funded by the EU from 2001 to 2004, for adding links from CABRI catalogues to other databanks available on-line and vice-versa. This effort included links from catalogues to Medline and to micro-organisms images and plasmids maps.

Within the EBRCN project, the identification and setting up of links to sequence databases was also foreseen. One of the most important molecular biology databanks is, as it is well known, the EMBL Data Library [4,5] that includes a large amount of publicly available nucleotide sequences as the result of a common effort by the European Bioinformatics Institute (EBI), the US National Center for Biotechnology Information (NCBI) and the Japanese National Institute of Genetics (NIG). The EMBL databank includes information on the sequence, its known features and related bibliography. The source material from where the sequence was determined is also reported. The EMBL databank also includes links to related records in external databases. It is therefore possible to define links from the sequences to the source material, when its description is also available on-line.

Automatic extraction from the sequence database of those identifiers that make reference to the relevant biological material is hampered by a number of shortcomings in EMBL entries. A first serious deficiency is that there is no consistent recording of the strain numbers that point out the individual cultures of the biological material from which the nucleotide sequences were obtained. According to the EMBL import specifications, this information should be stored in the qualifiers "isolate" or "strain" of the source feature, but the sequence deposit procedures do not strictly prohibit that depositors provide the strain label information within any of the other fields or – much more critical – do not provide this information at all.

Annotation and indexing problems, together with compilation errors, can also arise. This is particularly true because rules defined by culture collections for the naming of microbial strains have never been promoted and applied by scientists when writing their papers and by editors of databases when inserting data in their systems. Standard naming procedures define that strain numbers must consist of the collection acronym followed by a space and by either a number or an alphanumeric identifier. Of course, unique numbers/identifiers are given to each strain. Since these rules are not consistently followed, scientists often use different names for the same strain and this error is then propagated to databases contents.

References to biological resources in public knowledge bases seriously suffer from the "ID disparity" problem, which prevents integration of the data in a quick and efficient manner. It turns out that the same biological material may have multiple IDs in different databases, duplicate IDs in the same database and erroneous IDs caused by human error. The CABRI service turns out to provide a good solution for global ID integration by providing a single unique ID for each instance of the biological resources.

In this paper, we present the activity that was carried out in the frame of the EBCRN project for the identification of links between CABRI catalogues and the EMBL database of nucleotide sequences, the determination of valid cross-references between these databanks and the removal of false positives, i.e. cross-references resulting from annotation errors or synonymy. We also present results of this activity.

## Methods

### Identification of links

CABRI network service is based on an implementation of the Sequence Retrieval System (SRS), one of the most widespread and used search engine for molecular biology databases [6]. CABRI catalogues have been implemented with SRS by first comparing the data structures and contents of all collections' databases and then defining common data sets, unique for the different kind of resources. Guidelines for data input and authentication have also been defined and agreed upon. These often include references to common data sets and vocabularies [7]. Finally, collections have submitted their catalogues in a common flat file format for inclusion in the SRS site.

The EMBL database has also been implemented with SRS in many sites. Related configuration files are distributed with the software, so that the task of implementing an SRS site for accessing EMBL locally is relatively trivial.

Information on the biological source that was used for the determination of the sequence is included in the EMBL record within the Features Table (FT) section. This information is inserted as it is given by the submitter scientist, without checking for the correctness of the strain identifier. This implies different possible names for the same strain number, e.g., with or without spaces and other usual delimiters such as the dash or the slash characters.

Since SRS has internal features for linking those databases which are available in the same site, it therefore is the natural milieu for the determination of reciprocal links. These links are automatically defined by SRS by comparing index keys of relevant fields in involved databases: all records having at least one common index key are linked between them. This kind of links is particularly useful when linking databases that do not have direct ID based links among them, but share common data sets or vocabularies.

As anticipated, in our case strain numbers are not written in a consistent way. Moreover, it may happen that index keys are defined in different databases by using different methods, depending on the meaning it is assigned to the information in that context. In CABRI catalogues, strain numbers are indexed as unique keys. Instead, in EMBL they are indexed by single words. These different input and indexing strategies lead to incompatible index keys.

The strain number "LMG 6923" (the type strain of *Bacillus cereus*) would be indexed both as "LMG" and as "6923" in the EMBL database, while the unique key "LMG 6923" would be created for CABRI catalogues.

New indexing procedures must therefore be implemented in order to obtain uniform keys that can be used for establishing SRS links. It must also be taken into account that indexes in the EMBL database are not defined for each field. In our case, the EMBL index for strain numbers includes keys that are determined by scanning description texts of all FT fields.

A purpose SRS site, having both EMBL and CABRI catalogues, has been set up [8]. Indexing procedures for both EMBL source field and CABRI strain number field were adapted to the project's needs, thus avoiding the annotations and indexing problems that were previously mentioned.

Indexing scripts were modified so that index keys for CABRI unique identifiers and for source identifiers in the EMBL database share the same syntax and, in particular, only one index key is registered for each strain number where all spaces and special characters are removed.

Changes were carried out in the syntax definitions of both EMBL and CABRI catalogues. These definitions are written in the Icarus language and are used by SRS for the creation of databases' indexes. The changes included a redefinition of some regular expressions and the addition of a rule allowing for a special elaboration of only those strings in the strain field of the feature table that define the source name.

This made it possible to use the SRS native linking features to identify relationships between EMBL and CABRI records. EMBL (version 80) has been implemented in the SRS site together with CABRI catalogues (versions 2004.1). SRS links have been defined between EMBL and eleven catalogues of microbial strains. Links have then been automatically determined by SRS by comparing indexes of free text descriptions in EMBL Features Table section and indexes of strain numbers in CABRI catalogues (see table 2).

### Determination of valid cross-references

Results of the automatic identification of links were checked by comparing organism names as reported in the EMBL database and scientific names as reported in CABRI catalogues. To this end, purpose SRS views were created. This allowed for the downloading of lists of all links. These were then divided in three sub-lists including, respectively, valid, dubious and invalid links. We considered as valid all those links between records having identical strain numbers and organisms/scientific names. We defined as dubious all those links where organisms and scientific names differed, but could be checked for synonyms of names, alternate names and previous names. Dubious links were submitted for further evaluation to collections' staffs. Finally, we defined as invalid all those links where names were different and clearly not coherent (e.g., bacteria strains versus human sequences). These mainly originated from synonymy in biomedical terminology.

For bacterial strains, correctness of the cross-references was additionally validated by checking links to the EMBL records available in an integrated strain database. This database was obtained through the application of a software tool that automatically parses the complete EMBL database for extraction of all information that could represent a strain number. For the recognition of valid strain numbers in the EMBL records, this tool extracts instances of a regular expression that are composed of a unique acronym assigned to one of the culture collections mentioned in the directory of the World Data Centre for Microorganisms (WDCM; [9]), followed by a substring of numerical characters. In addition, the regular expression is insensitive both for any white space and for a selection of special characters that are located in between the acronym and the numerical substring. Acronym comparisons are treated in case insensitive mode. This way, the software tool takes into account the syntactic variability encountered in the use of strain numbers in the EMBL database. Each instance thus found is validated by a consultation of the Integrated Strain Database developed by Dawyndt et al. [10], where strain numbers that could not be automatically retrieved undergo a manual evaluation based on both the taxonomic identification and the literature information associated to the EMBL records. Similarly, the resolution of missing labels requires a time-consuming manual lookup within the literature references that are associated to the sequence records. This task is set up as a perpetual activity of the researchers from the Laboratory of Microbiology in Ghent, who are routinely working with the public sequence database during their research activities. As a result, the public sequence database is continuously enriched as a collaborative effort of the whole research community of the laboratory. At present, the attained success rate of this operation was that only 13,636 (10.4%) of these sequence records have been successfully linked in the way described above. Although a vast number of the currently unlinked records concern sequences related to uncultured bacterial strains, this experience from working with the EMBL sequence database is that still a significant number of the unlinked records can be manually linked, at the cost of a time-consuming lookup process.

Cross-references between EMBL accession numbers and CABRI strain numbers have been submitted to EBI for inclusion in the EMBL Data Library.

### The Interconnected Biological Resource Database

A new database, the Interconnected Biological Resources Database (IBRD) [11], was set up. IBRD is a compilation of essential information of all CABRI resources. Identification and Name data have been selected from each catalogue and inserted into the IBRD. Each IBRD record includes also a link to the original CABRI record.

IBRD is meant as a unique bridge between EMBL and CABRI catalogues: links from EMBL can be established to IBRD on the basis of the unique identifier, without having to specify the catalogue name. Since this information is included in the IBRD record, CABRI catalogues' records can then be reached with one further click.

## Results

As of EMBL version 81, more than 67,500 links to CABRI catalogues are available in the EMBL Data Library and distributed with it. The number of cross-references for each CABRI catalogue is shown in table 3.

HTML links can therefore be established from any implementation of the EMBL database to the CABRI network service either through the IBRD or directly to one defined CABRI catalogue. In the former case, the link to a generic resource identified by a determined strain number would be: http://www.cabri.org/CABRI/srs-bin/wgetz?+-page+qResult+-e+[IBRD:'strain_number'] while in the latter case the same resource in a defined catalogue would be linked to as: http://www.cabri.org/CABRI/srs-bin/wgetz?+-page+qResult+-e+[catalogue:'strain_number']

In both cases, spaces in strain numbers must be substituted by the string of characters "%20". It is also trivial to establish further links to CABRI from all those databanks that are available through SRS and have links to/from EMBL.

The procedures that have been used for the identification of links are executed whenever either EMBL or CABRI catalogues are updated in the purpose SRS site. Validation of these cross-references and submission of lists to EBI presently are almost manual procedures and the support from curators of culture collections is needed. Currently, we plan to update lists of valid cross-references every EMBL even version, that is twice a year.

## Conclusion

Since biological resources are essential tools for biomedical research today, we implemented a procedure for adding links between CABRI catalogues and the EMBL nucleotide sequence database. These links constitute an improved access to micro-organisms of certified quality and can produce positive effects on biomedical research. Further links between CABRI catalogues and bioinformatics databases can now easily be defined by using these cross-references, since the EMBL Data Library is deeply connected with many other databases in the bioinformatics network environment.

Linking genetic information incorporated within the public sequence databases onto information about the natural resources from which the DNA was extracted, and vice versa, may thus stand model for the integration of many other databases containing empirical data on the material kept in the biological resource centres. And notwithstanding the perpetual curation efforts that are required for checking the quality of the data involved in the unification process, the integration of biological data built upon solid cross-referencing schemes will offer a smart response to resolve for inconsistent data by avoiding the necessity of data replication.

## Figures and Tables

**Table 1:** Centres participating in CABRI and related resources type and number.

	**DNA probes**	**Bacteria strains**	**Fungi & yeasts strains**	**Plasmids**	**Phages**	**Human and banimal cell lines**	**Hybridomas**	**HLA typed B lines**	**Plant cells**	**Plant cell viruses**
**BCCM**		11,912	15,865	681						
**CABI**		243	11,677							
**CBS**		1,038	31,746							
**CIP**		7,038								
**DSMZ**		8,366	2,296	284	89	526			476	426
**ECACC**	261					956	397	238		
**ICLC**						237				
**NCCB**		4,609		396	69					
**NCIMB**		6,986			69					
**Total**	261	40,192	61,584	1,361	227	1,719	397	238	476	426

BCCM (Belgian Co-ordinated Collections of Micro-organisms, Brussels); CABI (CABI Bioscience, Egham); CBS (Centraalbureau voor Schimmelcultures, Utrecht); CIP (Collections de l'Institut Pasteur, Paris); DSMZ (Deutsche Sammlung von Mikroorganismen und Zellkulturen, Braunschweig); ECACC (European Collection of Cell Cultures, Salisbury); ICLC (Interlab Cell Line Collection, Genova); NCCB (The Netherlands Culture Collection of Bacteria, Utrecht); NCIMB (National Collections of Industrial, Marine and Food Bacteria, Aberdeen)

**Table 2:** EMBL to CABRI links determined automatically by SRS.

**From**	**Entries**	**To**	**Entries**	**Links Total**
EMBL	892	BCCM/LMG Bacteria strains	666	898
EMBL	5	CBS Bacteria strains	4	5
EMBL	4,246	DSMZ Bacteria strains	1,825	4,246
EMBL	349	NCIMB Bacteria strains	206	349
EMBL	830	CIP Bacteria strains	424	830
EMBL	28	BCCM/IHEM Fungi and Yeasts strains	22	28
EMBL	166	BCCM/MUCL Fungi and Yeasts strains	133	166
EMBL	10,481	CBS Filamentous fungi strains	2,181	10,488
EMBL	55,866	CBS Yeasts strains	979	55,874
EMBL	305	CABI Bioscience Fungi strains	206	305
EMBL	50	DSMZ Fungi and Yeasts strains	35	50

. The reported figures represents the number of EMBL records having a link to the corresponding CABRI catalogue version 2004.1 (second column) and of CABRI records having a link to EMBL version 80 (fourth column). The fifth column reports the total number of links.

**Table 3:** EMBL 81 to CABRI cross-references.

CBS Yeasts catalogue (huge records)	51,103
CBS Filamentous fungi catalogue (huge records)	7,116
DSMZ Bacteria catalogue	2,618
CBS Filamentous fungi catalogue	2,551
CBS Yeasts catalogue	1,880
BCCM/LMG Bacteria catalogue	799
CIP Bacteria catalogue	546
NCIMB Bacteria catalogue	336
BCCM/MUCL Fungi and yeasts catalogue	119
DSMZ Fungi catalogue	31
BCCM/IHEM Fungi and yeasts catalogue	18
Total	67,528

The reported figures represent the number of records in EMBL version 81 having a cross-reference to the corresponding CABRI catalogue. The term 'huge records' with some catalogues names makes reference to a slightly different, separate analysis that has been carried out for those strains having a huge number of references in the EMBL data library.
